# Low level of HIV-1C integrase strand transfer inhibitor resistance mutations among recently diagnosed ART-naive Ethiopians

**DOI:** 10.1038/s41598-023-33850-4

**Published:** 2023-04-21

**Authors:** Mulugeta Kiros, Dessalegn Abeje Tefera, Henok Andualem, Alene Geteneh, Abebech Tesfaye, Tamirayehu Seyoum Woldemichael, Eleni Kidane, Dawit Hailu Alemayehu, Melanie Maier, Adane Mihret, Woldaregay Erku Abegaz, Andargachew Mulu

**Affiliations:** 1 Department of Medical Laboratory Science, College of Medicine and Health Sciences, Aksum University, Aksum, Ethiopia; 2 Armauer Hansen Research Institute, Addis Ababa, Ethiopia; 3 Department of Medical Laboratory Science, College of Medicine and Health Sciences, Debre Tabor University, Debre Tabor, Ethiopia; 4 Department of Medical Laboratory Science, College of Health Sciences, Woldia University, Woldia, Ethiopia; 5 The Ethiopian Public Health Institute, Addis Ababa, Ethiopia; 6 Department Virology, Institute Medical Microbiology and Virology, Leipzig University, Leipzig, Germany; 7 Department of Microbiology, Parasitology, and Immunology, School of Medicine, Addis Ababa University, Addis Ababa, Ethiopia

**Keywords:** Microbiology, Medical research

## Abstract

With the widespread use of Integrase strand transfer inhibitors (INSTIs), surveillance of HIV-1 pretreatment drug resistance is critical in optimizing antiretroviral treatment efficacy. However, despite the introduction of these drugs, data concerning their resistance mutations (RMs) is still limited in Ethiopia. Thus, this study aimed to assess INSTI RMs and polymorphisms at the gene locus coding for Integrase (IN) among viral isolates from ART-naive HIV-1 infected Ethiopian population. This was a cross-sectional study involving isolation of HIV-1 from plasma of 49 newly diagnosed drug-naive HIV-1 infected individuals in Addis-Ababa during the period between June to December 2018. The IN region covering the first 263 codons of blood samples was amplified and sequenced using an in-house assay. INSTIs RMs were examined using calibrated population resistance tool version 8.0 from Stanford HIV drug resistance database while both REGA version 3 online HIV-1 subtyping tool and the jumping profile Hidden Markov Model from GOBICS were used to examine HIV-1 genetic diversity. Among the 49 study participants, 1 (1/49; 2%) harbored a major INSTIs RM (R263K). In addition, blood specimens from 14 (14/49; 28.5%) patients had accessory mutations. Among these, the M50I accessory mutation was observed in a highest frequency (13/49; 28.3%) followed by L74I (1/49; 2%), S119R (1/49; 2%), and S230N (1/49; 2%). Concerning HIV-1 subtype distribution, all the entire study subjects were detected to harbor HIV-1C strain as per the IN gene analysis. This study showed that the level of primary HIV-1 drug resistance to INSTIs is still low in Ethiopia reflecting the cumulative natural occurrence of these mutations in the absence of selective drug pressure and supports the use of INSTIs in the country. However, continues monitoring of drug resistance should be enhanced since the virus potentially develop resistance to this drug classes as time goes by.

## Introduction

Globally antiretroviral therapy (ART) has been scaled up with an estimated 26 million people accessing at the end of 2020 that led to the dramatic decrease of mortality and morbidity due to Human Immunodeficiency virus-1 (HIV-1) associated diseases^1,2^. Despite this, however, the emergence of HIV-1 pre-treatment drug resistance (PDR) is posing a threat to the success of ART^3^. PDR reduces the treatment options available to newly HIV-1 infected patients, and it is associated with an increased risk of virological failure^2^. Therefore, surveillance of drug resistance mutations (RMs) among ART-naive patients is critical in optimizing ART efficacy^4^.

Due to an increasing trend of HIV drug resistance (HIVDR) to non-nucleoside reverse-transcriptase inhibitor (NNRTI), nucleoside reverse transcriptase inhibitor (NRTI), and protease inhibitors (PI), integrase strand transfer inhibitor (INSTI)-based regimens are now recommended as first-line combination ART in several updated treatment guidelines^4^. Hence, they have become an essential component of ART in use in many countries including Ethiopia. These latest antiretroviral (ARV) drug class works via the inhibition of DNA strand transfer and are clinically effective against HIV-1 strains that had previously exhibited resistance-associated mutations against other ARV drugs^5^. To date, five INSTIs are approved for clinical use by the US food and drug administration, namely, raltegravir (RAL), elvitegravir (EVG), bictegravir (BIC), dolutegravir (DTG), and cabotegravir (CAB)^4–7^. Among these INSTI drugs, DTG, which is a second generation drug, is preferable due to some notable advantages including having a limited or no-cross resistance to early generation INSTI drugs, and being responsible for the higher inhibition potency^8^. However, drug RMs like Q148H/R and G140S in combination with mutations L74I/M, E92Q, T97A, E138A/K, G140A, or N155H are associated with 5-fold to 20-fold reduction in DTG effectiveness^9^. In addition, the R263K, which is the most commonly DTG-selected mutation, is associated with a 2-fold susceptibility reduction when it occurs alone^10,11^.

DTG was introduced as a preferred first-line drug combination in Ethiopia in 2018^12^. However, data concerning INSTI drug RMs is still limited^13^ in the country. The aim of this study was thus to generate updated information regarding the prevalence of INSTI associated drug RMs among ART-naive Ethiopian populations before the introduction of DTG.

## Materials and methods

### Study population

This is a cross-sectional health facility-based study involving drug-naive HIV-1 infected study participants. Study participants who were asymptomatic at the time of recruitment, above the age of 18, and willing to take part in the study were sequentially enrolled. This research did not include pregnant women, those with known chronic conditions, or anyone who had ever used ART. For detailed patients screening and enrollment, please refer to our previous publication^14^. Accordingly, forty-nine volunteered adult (≥ 18 years old) study participants from voluntary VCT centers in Addis Ababa were consecutively enrolled in this study from June to December 2018. Blood samples (10 ml) were collected at the time of HIV diagnosis before initiation of ART for genotypic assay of the IN gene region from these participants using an EDTA tube. Plasma was then separated by centrifugation for 10 min at 3000 rpm and stored in deep freeze (− 80 °C) till required for laboratory investigation.

### Nucleic acid extraction, PCR amplification, and sequencing

Viral RNA extraction and viral load were performed consecutively from 200 μL of thawed participants' plasma sample input using the Abbott Real-time HIV-1 M2000 system (Abbott Laboratories, Abbott Park, USA). For sequencing purpose, cDNA was synthesized from the extracted RNA in a 20 microliter (µL) reaction mixture using Superscript IV Reverse Transcriptase enzyme and HIVpcrRev1 primer (Table 1). The mixture constituted; 1 µL of HIVpcrRev1 outer primer, 1 µL of dNTPs, 1 µL of DDT, 1 µL of RiboLock, 4 µL of 5X superscript IV buffer, 1 µL of superscript IV (Invitrogen Carlsbad, CA, USA), 5 µL of RNA, and 6 µL of molecular grade water. The thermal cycling for this cDNA synthesis was; 50 °C for 1 h^13^.

**Table 1:** List of in-house primers used for Integrase gene PCR amplification and Sanger sequencing [Adopted from Reference 13].

Name	Sequence	Position*
HIVpcrRev1	5′TGGGATGTGTACTTCTGAACTTA3′	5192–5214
HIVpcrFor1	5′AAAGGAATTGGAGGAAATGAAC3′	4167–4188
HIVpcrRev2	5′CCTGCCATCTG TTTTCCATA3′	5040–5059
HIVpcrFor2	5′GAAATGAACAAGTAGATAAATTAGTAAG3′	4180–4204

*All positions are matched to HIV-1 HXB2 (GenBank Accession number K03455).

This was followed by a first-round PCR, which was performed in a 50 µL reaction mix composed of 1 µL of each outer primer (HIVpcr For1 and HIVpcr Rev1; Table 1), 27.8 µL of nuclease‐free water, 10 µL of 5X GoTaq buffer, 4 µL of 25 mM MgCl_2_, 1 µL of 10 mM dNTPs, 0.2 µL of GoTaq polymerase (Promega, USA), and 5 µL of cDNA. The PCR cycling conditions were 98 °C for 2 min followed by 39 cycles of 10 s at 98 °C, 25 s at 64 °C, 40 s at 72 °C, and a final extension of 72 °C for 5 min^13^.

The DNA product from the first round PCR was then re-amplified by a nested PCR. This nested PCR was carried out in another 50 µL reaction mix utilizing 1 µL of the first-round PCR product, 1 µL of each inner primer (HIVpcr For2 and HIVpcr Rev2; Table 1), 31.8 µL of nuclease‐free water, 10 µL of 5X GoTaq buffer, 4 µL of 25 mM MgCl_2_, 1 µL of 10 mM dNTPs, and 0.2 µL of GoTaq polymerase (Promega, USA). This final re-amplified PCR product (793 bp) was verified by agarose gel electrophoresis (using 1.5% agarose gel) and was then purified consecutively using the GenepHlow™ Gel/PCR Kit (Geneaid biotech ltd., Taiwan), following the manufacturer’s instruction. Purified amplicons were then sequenced with Sanger DNA sequencing using the BigDye Terminator v3.1 Cycle Sequencing Kit (Applied Biosystems), and run on the ABI PRISM® 3500 xL automated Genetic Analyzer. At each step of the laboratory procedures, both positive and negative controls were incorporated in order to keep the procedures quality assurance.

### Primary INSTI resistance mutation analysis and HIV-1 subtype determination

Sequences were first collected from the ABI PRISM® 3500 xL automated Genetic Analyzer (Applied Biosystems) and transferred to separate personal computer. Sequence edition and alignment were then made using Geneious prime® software v.2020.2.1 (https://www.geneious.com/academic/). The quality of the sequence data was checked using an online sequence quality control tool found in the Los Alamos HIV sequence database (http://hiv-web.lanl.gov). Calibrated Population Resistance (CPR) tool version 8.0, which is available at Stanford University HIV Drug Resistance Database (HIVdb) (http://cpr.standford.edu/cpr/index.html) was used to assess the drug RMs. The susceptibility of HIV to ARV drugs was determined using the HIVdb program (http://hivdb.stanford.edu).

All sequences were thoroughly examined for the presence of primary mutations, nonpolymorphic [NoP] and polymorphic mutations associated with resistance to INSTI. Each aminoacid prevalence at each IN position was calculated and compared to the HIV-1 subtype B reference sequence (GenBank accession number: K03455). For this study, NoP was defined as substitutions within the HIV-1 IN that occurred in ≥ 1% of the sequences. Positions with ≥ 20% substitutions were considered as highly polymorphic, while those with ≤ 0.5% substitution were considered highly conserved.

HIV-1 subtype determination was performed using two online HIV-1 subtyping tools; the REGA version 3 online HIV-1 subtyping tool from the Stanford HIVdb (http://hivdb.stanford.edu) and the jumping profile Hidden Markov Model (jpHHM) at GOBICS (http://jphmm.gobics.de/). The subtypes were also further confirmed by phylogenetic analysis using reference sequences from Los Alamos National Laboratory HIV Sequence Database (http://hiv-web.lanl.gov). The analysis was made using Molecular Evolutionary Genetics Analysis (MEGA) v.10.0.5 software^15^.

### Statistical analyses

Epi data v3.1 software was used for data entry while SPSS version 25.0 (SPSS Inc. the United States) was used for analysis. A logistic regression model was used to determine the presence of any associations between drug RMs and sociodemographic variables. Thus, variables having a *p* value < 0.05 were considered to have a statistically significant association. All isolates are deposited in GenBank with accession number MW560010 to MW560058.

### Ethics approval and consent to participate

This study was approved by the institutional ethical review committee of the Armauer Hansen Research Institute (Protocol Number: PO16/18). All participants provided their written informed consent to participate in this study and that this study was conducted following the declaration of Helsinki.

## Results

### Characteristics of the study population

Near full-length HIV-1 IN region, (263 codons) of the 49 study participants were successfully sequenced and analyzed. Of these, 59.2% (n = 29) were females (Table 2). The mean ± SD age of the participants was 32 ± 1.2 years ranging from 20 to 57 years old. The median (IQR) viral load was 69,625 copies/mL (32,219–208,299). The detailed demographic and clinical information of the study participants is summarized in Table 2.

**Table 2:** Participants’ sociodemographic and virological characteristics.

Characteristics		Frequency (N (%))	Individuals with major INST RM (N)
Sex	Male	20 (40.80	0
	Female	29 (59.2)	1
Age category	18–28	17 (34.7)	1
	29–38	22 (44.9)	0
	39–48	7 (14.3)	0
	> 49	3 (6.1)	0
Baseline viral load (Copies/ml)	< 1000	1 (2.0)	0
	1000–10,000	5 (10.2)	0
	10,001–100,000	22 (44.9)	0
	> 100,000	21 (42.9)	1
Occupation	Unemployed	19 (38.8)	1
	Employed	30 (61.2)	0
Marital status	Married	19 (38.8)	0
	Single	15 (30.6)	1
	Divorced	9 (18.4)	0
	Widowed/widower	6 (12.2)	0
Educational status	No schooling	8 (16.30	0
	Primary	13 (26.5)	1
	Secondary	18 (36.7)	0
	College (diploma)	4 (8.2)	0
	University degree	6 (12.2)	0

*INSTI* Integrase strand transfer inhibitor, *RM* Resistance mutation.

### The level of primary INSTI resistance mutation and HIV-1 Subtype distribution

**Figure 1:**
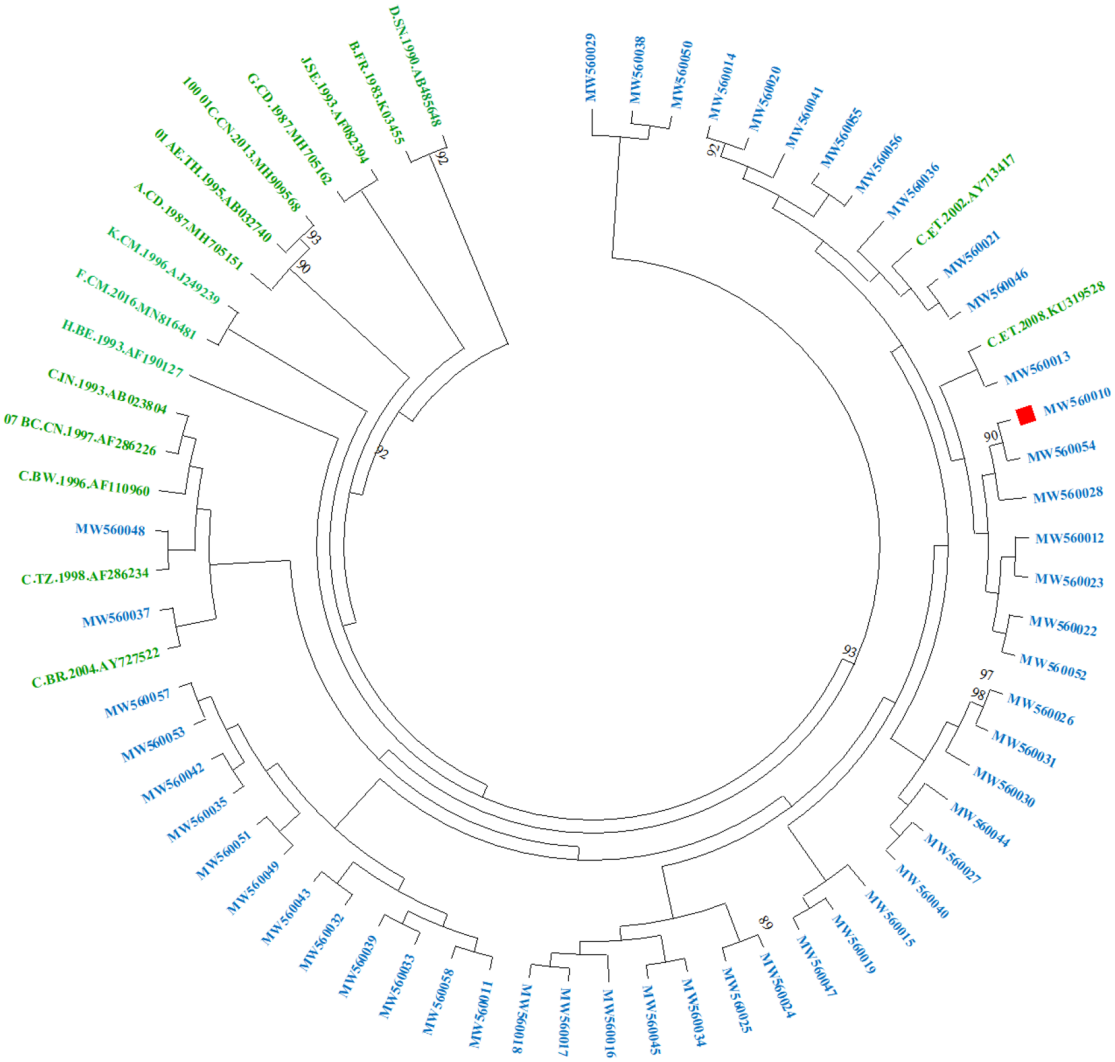
Maximum-likelihood phylogenetic tree of the HIV-1 integrase sequences from the 49 drug-naive HIV-infected individuals. The ML tree was constructed using MEGA version 10.0.5 with the Kimura 2 parameter. Bootstrapping was performed with 1,000 replicates and only those that have a bootstrap value > 70% are shown on the tree. HIV-1 reference sequences (A—K and CRFs) that were retrieved from the HIV-1 LANL HIV Sequence Database (green color) are indicated by subtype followed by the corresponding country name, year, and accession number. All of the sequences from this study that are indicated in blue color (MW560010–MW560058) were clustered with HIV-1 subtype C strain with a bootstrap value of 93%. The one sequence (MW560010) that harbored a major INSTI RM R263K is shown with a red rectangular node. *INSTI* Integrase strand transfer inhibitor, *LANL* Los alamos national library, *MEGA* Molecular evolutionary genetics analysis, *ML* Maximum likelihood.

**Figure 2:**
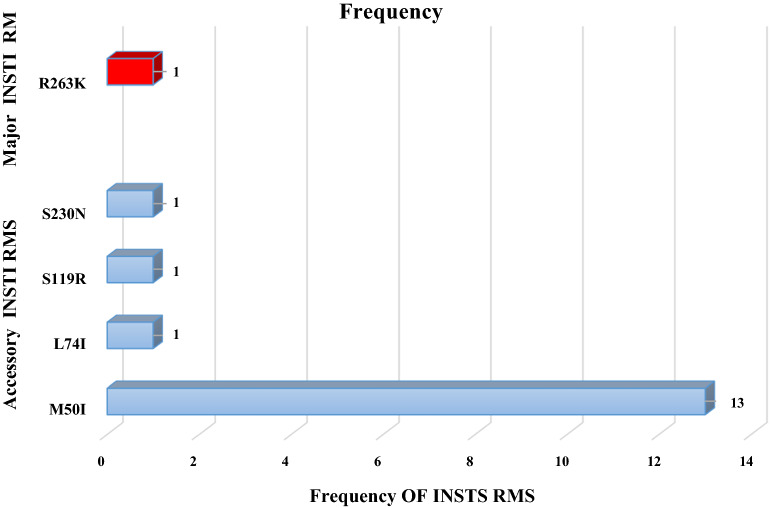
Frequency of major and accessory INSTI-RMs detected. The major INSTI-RM was R263K while the accessory RMs were M50I, L74I, S119R, and S230N. *RMs* Resistance mutations.

As per the INSTI RMs analysis, one (n = 1/49; 2%) participant harbored one major INSTIs RM R263K (Fig. 2.). Concerning the HIV-1 subtype distribution, both the REGA version 3 online HIV-1 subtyping tool and the jumping profile Hidden Markov Model from GOBICS indicated that the entire 49 subjects were harboring HIV-1C virus as per the IN region analysis. This was further confirmed by a maximum likelihood phylogenetic analysis along with the reference sequences (A–K) and CRFs from the Los Alamos National Library database (www.lanl.gov) (Fig. 1.). As indicated in the phylogenetic tree, sequences from this study with GenBank accession number (MW560010 to MW560058) were clustered with HIV-1C with a bootstrap value of 93% (Fig. 1).

### Accessory INSTI RMs and/or polymorphisms

Besides the major INSTI RM observed, 14 (14/49; 28.5%) patients in the current study harbored at least one accessory RMs. Among these, almost 93% of the accessory mutations observed were M50I (13/49; 28.3%) followed by L74I (1/49; 2%), S119R (1/49; 2%), and S230N (1/49; 2%) (Fig. 2.). Two patients had two simultaneous accessory RMs (M50I and S119R; M50I and S230N). Furthermore, other naturally occurring polymorphisms that indicate variability between HIV-1 subtypes were also observed.

## Discussions

INSTIs have rapidly become an important class in the arsenal of ARV drugs due to their higher genetic barrier^6,16^. The world health organization (WHO) recommended one of the INSTI drugs, DTG, as a preferred first-line ART in 2017^17^. This drug classes is now becoming the best choice in many countries due to an increase in resistance rates against NNRTIs, NRTIs as well as PIs in the past years^18^.

In Ethiopia, where no routine genotyping testing nor routine drug resistance surveillance is present, this drug was introduced in 2018 following the aforementioned WHO recommendation^12^. However, studies on primary HIV-1 drug resistance related to these drugs are limited in the country. So far, only a single study that indicated a lack of a major INSTI RM was reported^13^. Therefore, the current study was intended to provide an update on INSTI RM prevalence among untreated HIV-1 infected people in Ethiopia after 10 years of a previous study^13^. Accordingly, this study found out that the prevalence of baseline major INSTI RM among untreated patients is 2%. This observation of low level resistance is in line with the previous studies from Ethiopia^13^, Brazil^19^, South Africa^18,20^, India^21^, Cameroon^22^, Europe^23^, and Taiwan^4^.

The only major INSTI RM detected in the current study is a non-polymorphic mutation R263K that can be selected in vivo during a treatment with DTG and RAL, where it is capable of causing a 2 to 5-fold reduction in susceptibility to DTG, EVG, and RAL^6^. In addition, R263K has been observed to be selected in vitro under pressure with BIC and the INSTI investigational drug CAB^6^.

Compared to high-income settings, where it is primarily associated with subtype B-infected individuals treated with ABC/3TC/DTG, this R263K mutation, along with other DTG RMs, is reported to be more common in low-middle-income settings, where patients with treatment failure use an alternative NRTI drug class, leading to the accumulation of multi-NRTI resistance^24^.

**Figure 3:**
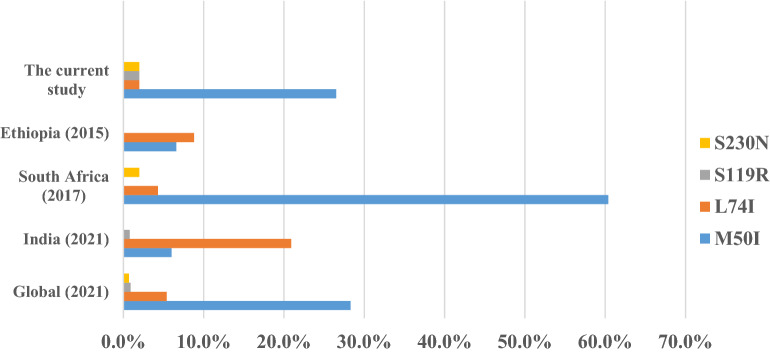
Comparision on accessory mutations.

In this study, we have also further detected accessory RMs that have little effect unless they present with other major mutations. The overall magnitude of these polymorphic accessory RMs was 28.5%, which is higher than detection rate in studies from Cameroon 8.1–10%^22,25^, India 10.1%^26^, and sub-Saharan Africa 8.7%^27^. Among the minor RMs detected in the current study, M50I was the most common accessory RMs (13 of the 14 (93%) minor mutations detected from 13/49 (28.3%) participants, which is in line with the magnitude of its global distribution^28^ (Fig. 3). Moreover, this detection rate is higher relative to the report from previous study in Ethiopia^13^, indicating the increases this variant's circulation in the country. M50I polymorphic mutation is selected in vitro by DTG and BIC in combination with R263K, a combination that appears to reduce DTG susceptibility^6^.

The other minor RMs detected were L74I (1/49; 2%), S119R (1/49; 2%), and S230N (1/49; 2%). L74M alone has minimal, if any, effect on INSTI susceptibility. However, they contribute to reduced susceptibility to each of the INSTIs when present in combination with major mutations^6^. S119R is a polymorphic mutation that is weakly selected by INSTIs usually in combination with several major INSTI-associated drug RMs. Alone, it has little, if any effect, on INSTI susceptibility^28^. While all these three accessory RMs were previously reported from other countries like South Africa^18^ and India^28^ in line with the current study, only the former (L74I) was detected previously in Ethiopia^13^. The overall comparative summary of these accessory mutations detected in the current study with HIV-1 isolates from untreated patients from South Africa^18^, India^28^, Ethiopia^13^, and worldwide^28^ is presented in Fig. 3.

Regarding the HIV-1 integrase diversity observed, this study found out that all 49 (100%) study subjects harbored HIV-1C subtype, which is in line with previous reports from Ethiopia. This confirms that the HIV-1 strain circulation in Ethiopia is still dominated by HIV-1C despite the early introduction of the clade^29^.

## Conclusions and recommendations

In conclusion, this study confirmed homogeneity in the circulating HIV-1 clade C and indicated that the level of primary HIV-1 resistance to INSTIs among treatment naive population is still low in Ethiopia, which supports the use of INSTIs in the country. However, the observation from this study does not support the need for performing INSTI resistance testing at baseline, as the prevalence is low. Nevertheless, further larger studies are necessary to assess the impact of accessory INSTI RMs on INSTI-based ART regimens.

## Data Availability

All nucleotide sequences from this study have been deposited in the Genbank (ncbi) repository with accession numbers from MW560010 to MW560058.

## Abbreviations

AHRI: Armauer hansen research institute
AIDS: Acquired immuno deficiency syndrome
ART: Antiretroviral treatment
ARV: Antiretroviral
HIV: Human immunodeficiency virus
HIVDR: HIV drug resistance
IN: Integrase
INSTI: Integrase strand transfer inhibitor
NRTI: Nucleoside reverse-transcriptase inhibitor
NNRTI: Non-nucleoside reverse transcriptase inhibitor
PCR: Polymerase chain reaction
PDR: Primary drug resistance
PI: Protease inhibitor
PR: Protease
RM: Resistance mutation
RT: Reverse transcriptase
NoP: Nonpolymorphic
WHO: World health organization
